# Early enzyme replacement therapy prevents dental and craniofacial abnormalities in a mouse model of mucopolysaccharidosis type VI

**DOI:** 10.3389/fphys.2022.998039

**Published:** 2022-09-21

**Authors:** Rohit Nagpal, Gina Georgi, Sarah Knauth, Carmen Schmid-Herrmann, Nicole Muschol, Thomas Braulke, Bärbel Kahl-Nieke, Michael Amling, Thorsten Schinke, Till Koehne, Julian Petersen

**Affiliations:** ^1^ Department of Osteology and Biomechanics, University Medical Center Hamburg-Eppendorf, Hamburg, Germany; ^2^ Department of Orthodontics, University of Leipzig Medical Center, Leipzig, Germany; ^3^ Department of Orthodontics, University Medical Center Hamburg-Eppendorf, Hamburg, Germany; ^4^ Department of Pediatrics, University Medical Center Hamburg-Eppendorf, Hamburg, Germany

**Keywords:** MPS VI, Arsbm/m mice, condyle, alveolar bone loss, craniofacial malformations

## Abstract

Mucopolysaccharidosis VI (MPS VI) is a hereditary lysosomal storage disease caused by the absence of the enzyme arylsulfatase B (ARSB). Craniofacial defects are common in MPS VI patients and manifest as abnormalities of the facial bones, teeth, and temporomandibular joints. Although enzyme replacement therapy (ERT) is the treatment of choice for MPS VI, the effects on the craniofacial and dental structures are still poorly understood. In this study, we used an Arsb-deficient mouse model (*Arsb* ^*m/m*^) that mimics MPS VI to investigate the effects of ERT on dental and craniofacial structures and compared these results with clinical and radiological observations from three MPS VI patients. Using micro-computed tomography, we found that the craniofacial phenotype of the *Arsb* ^*m/m*^ mice was characterized by bone exostoses at the insertion points of the masseter muscles and an overall increased volume of the jaw bone. An early start of ERT (at 4 weeks of age for 20 weeks) resulted in a moderate improvement of these jaw anomalies, while a late start of ERT (at 12 weeks of age for 12 weeks) showed no effect on the craniofacial skeleton. While teeth typically developed in *Arsb* ^*m/m*^ mice, we observed a pronounced loss of tooth-bearing alveolar bone. This alveolar bone loss, which has not been described before in MPS VI, was also observed in one of the MPS VI patients. Interestingly, only an early start of ERT led to a complete normalization of the alveolar bone in *Arsb* ^*m/m*^ mice. The temporomandibular joints in *Arsb* ^*m/m*^ mice were deformed and had a porous articular surface. Histological analysis revealed a loss of physiological cartilage layering, which was also reflected in an altered proteoglycan content in the cartilage of *Arsb* ^*m/m*^ mice. These abnormalities could only be partially corrected by an early start of ERT. In conclusion, our results show that an early start of ERT in *Arsb* ^*m/m*^ mice achieves the best therapeutic effects for tooth, bone, and temporomandibular joint development. As the MPS VI mouse model in this study resembles the clinical findings in MPS VI patients, our results suggest enzyme replacement therapy should be started as early as possible.

## Introduction

Mucopolysaccharidosis type VI (MPS VI or Maroteaux-Lamy syndrome) belongs to a group of lysosomal storage diseases characterized by enzymatic defects in lysosomal degradation of sulfated glycosaminoglycans (Valayannopoulos et al., 2010). The prevalence of MPS VI is estimated to be 1–9: 1,000,000, and the disease is inherited in an autosomal recessive manner. MPS VI patients have mutations in the *ARSB* gene, which codes for the enzyme arylsulfatase B (also called N-acetylgalactosamine-4-sulfatase). Since this enzyme is involved in the lysosomal degradation of the glycosaminoglycans dermatan sulfate and chondroitin 4-sulfate, these glycosaminoglycans accumulate in all tissues and organs in MPS VI (O'Brien et al., 1974). The first manifestation of MPS VI occurs in childhood. The most striking symptoms of the disease include dysplastic and degenerative changes in the entire skeleton, such as short stature, dysostosis multiplex, and degenerative joint changes (Valayannopoulos et al., 2010). The temporomandibular joints are also affected, contributing to the distinct craniofacial phenotype of the patients. (de Almeida-Barros et al., 2012). To date, enzyme replacement therapy (ERT) with recombinant human arylsulfatase B (rhARSB) is the treatment of choice for MPS VI (Chen et al., 2019). rhARSB is modified with mannose 6-phosphate (M6P) residues which mediate the M6P receptor-dependent uptake into cells (Sly et al., 2006). Although ERT can restore the enzymatic removal of sulfate groups from dermatan sulfate and chondroitin 4-sulfate, the therapeutic success of ERT varies from organ to organ. In the late 1990s, the first studies on the effect of ERT in a mouse model of the human MPS VI disease showed almost complete normalization and a decrease in the accumulation of GAGs in the tissues. Exceptions, however, were cartilage tissue and the cornea (Crawley et al., 1997). A rapid reduction of GAGs in the urine was also observed in clinical ERT studies (Harmatz et al., 2006). The stabilizing effect and safety of this therapy has been continuously confirmed in recent years (Harmatz et al., 2006; Harmatz et al., 2019). Animal studies (Auclair et al., 2006) and studies with affected twins and siblings have shown that the earlier the therapy is started, the better the prognosis and the success of the treatment (McGill et al., 2010; Furujo et al., 2011). In addition, we were able to demonstrate the positive effects of enzyme replacement therapy on the skeletal system using an Arsb-deficient mouse model (*Arsb* ^*m/m*^) (Pohl et al., 2018; Hendrickx et al., 2020). In these studies we also showed that the mandibular bones of *Arsb* ^*m/m*^ have typical exostoses at the mandibular rim that can be reduced by enzyme replacement therapy. However, the reasons for these exostoses, remained unclear. In addition, no analyses of the teeth or jaw joints have been reported so far in these mice.

In this study, we examined skulls of 24-week-old *Arsb* ^*m/m*^ mice and control mice using contact radiography, micro-computed tomography (μCT), and decalcified histology in comparison with craniofacial and dental findings from three MPS VI patients. In addition, to better understand the effect of ERT on facial bones, teeth, and temporomandibular joints, we analyzed 24-week-old *Arsb* ^*m/m*^ mice in which ERT was started at 4 or 12 weeks of age, respectively.

## Results

### Partial correction of the craniofacial phenotype of *Arsb* ^*m/m*^ mice by ERT

**FIGURE 1:**
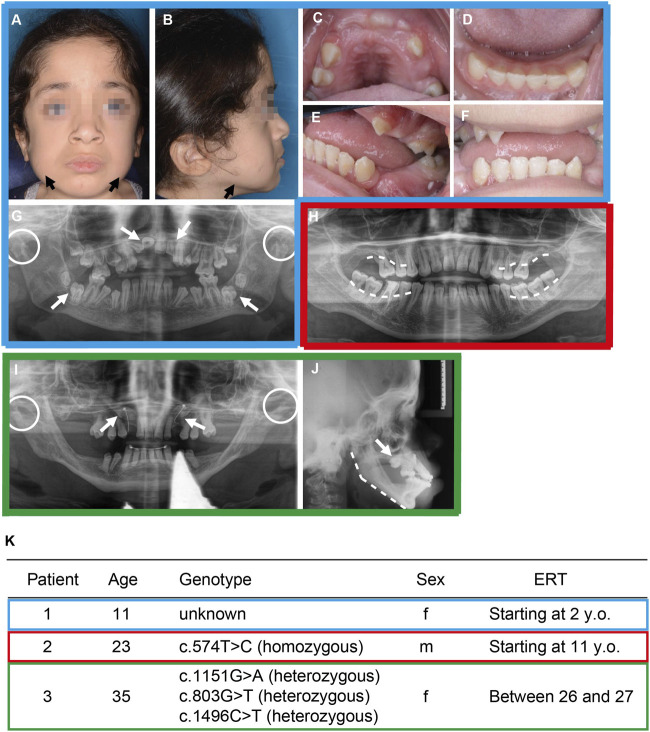
Craniofacial and dental pathologies of patients with Mucopolysaccharidosis Type VI. **(A–G)** Patient #1 at 11 years treated with ERT (Naglazyme, BioMarin Pharmaceuticals) starting from the age of 2. **(H)** Patient #2 at 23 years treated with ERT starting at the age of 11 years. **(I–J)** Patient # 3 at 35 years treated with ERT between the age of 26 and 27. **(A–B)** Extraoral photographs display the MPS VI–typical facial coarsening with mandibular bone exostoses at the jawline (black arrows). **(C–F)** Intraoral photographs show gingival hyperplasia and delayed anterior teeth eruption, which normally erupts in the age between 6 and 9 years. **(G)** Panoramic radiograph image showing displaced and retained incisors and dentigerous cysts of the first mandibular molars (white arrows). Bleeding on probing was observed in periodontal screenings. Furthermore, condylar hypoplasia is marked with white dotted circles. **(H)** Panoramic radiograph image reveals progressive maxillar and mandibular horizontal bone loss (white dashed lines). In periodontal screenings, probing depths over 6 mm were measured. Teeth 37 and 47 were extracted afterward due to increased mobility. Mandibular condyles are not shown in this image. **(I)** Panoramic radiograph image showing retained cuspids and orthodontic traction chains (white arrows). Condylar hypoplasia is marked with white dotted circles. **(J)** Lateral cephalometric radiograph displaying severe vertical facial growth including flat mandibular angle (white dashed line). Retention of the cuspids is shown from a lateral view (white arrow). **(K)** General information of the three patients including genomic modification within the ARSB gene. Here, **(C)**574T>C, corresponding to p. C192R, and **(C)**1151G>A (p.S384N) are pathological (Lin et al., 2008; Tomanin et al., 2018). The mutation **(C)**1496C>T (p.L499P) is probably also pathological, since an L498P substitution impairs the a-helix structure of ARSB and thus probably its function. (Litjens et al., 1996). However, we additionally identified a previously unknown mutation c803G>T (p.G268V), which was not previously described in the literature (Panel 1K).

MPS VI patients show typical changes in facial morphology, collectively described as coarsening facial features (Galimberti et al., 2018). By investigating extraoral photographs and panoramic radiograph images of three Mucopolysaccharidosis Type VI patients who had been treated with ERT (Naglazyme, BioMarin Pharmaceuticals) at different time points (#1 age 11 started treatment at the age of 2, #2 age 23 started treatment at the age of 11 and #3 age 35 was treated only 1 year between 26–27 of age) we observed similar dental and craniofacial phenotypes as described in the literature (Figure 1). In particular, the facial photographs of patient #1 showed coarsening of the facial features, with pronounced jaw muscles (Figures 1A,B), even though this patient had been receiving ERT since the age of 2 years.

**FIGURE 2:**
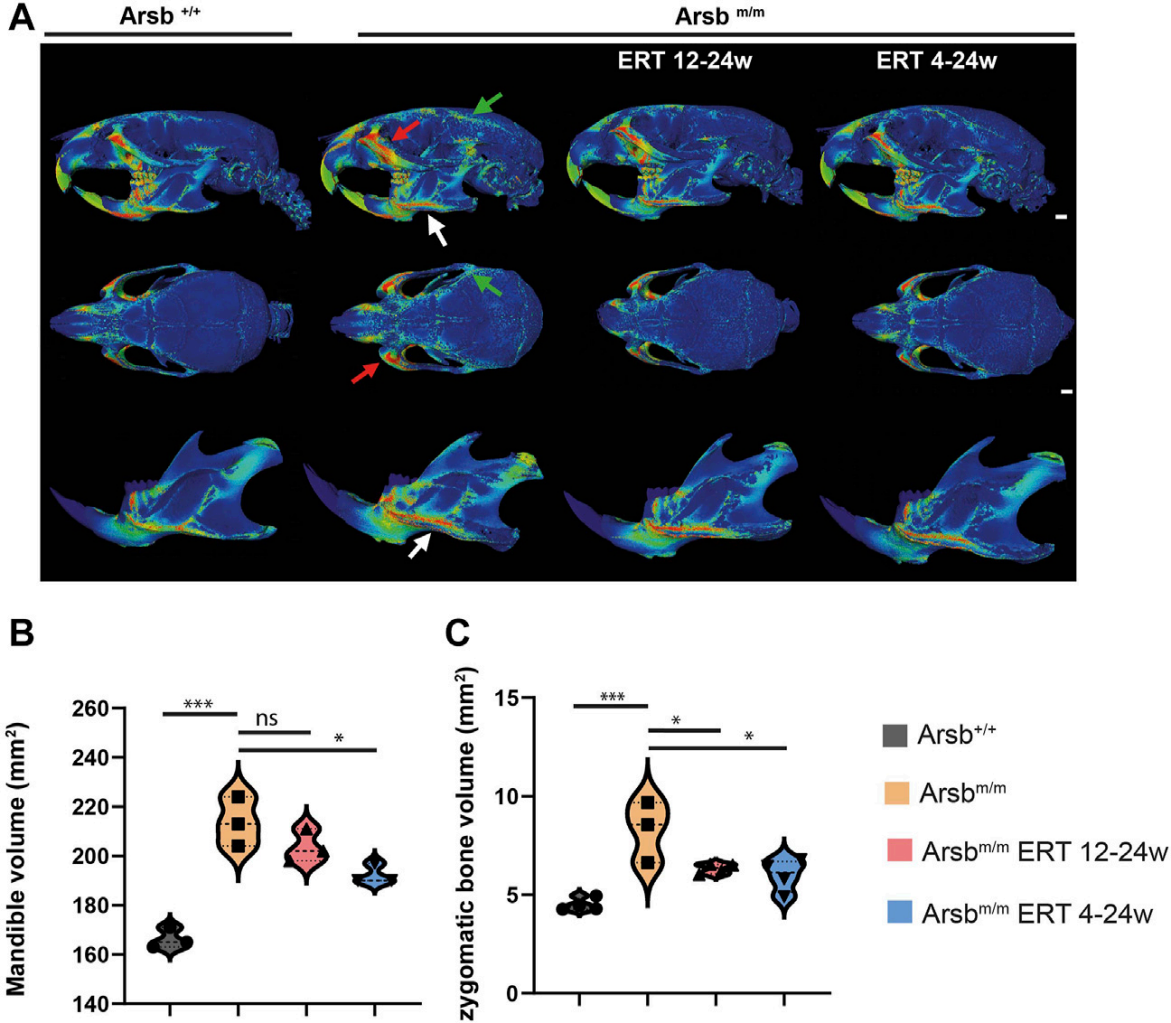
Bone exostoses on the craniofacial skeleton of MPS VI mice. **(A)** Analysis of wall thickness from micro-CT scans of the skulls of 24-week-old control (*Arsb* ^*+/+*^), MPS VI (*Arsb* ^*m/m*^), and MPS VI mice in which enzyme replacement therapy was initiated after 12 weeks of age (*Arsb* ^*m/m*^ ERT 12-24w representing late-start ERT) and after 4 weeks of age (*Arsb* ^*m/m*^ ERT 4-24w representing early-start ERT). View from the side (upper panel), overview (middle panel), and separated mandible (lower panel). *Arsb* ^*m/m*^ mice show bone exostoses at the marked locations: lower margin of the mandibular body (white arrow), zygomatic arch (red arrow), and temporal line (green arrow). These exostoses are less pronounced in *Arsb* ^*m/m*^ mice with ERT. Scale bar = 1 mm. **(B)** Quantification of mandibular bone volume. **(C)** Quantification of the zygomatic bone volume. *p < 0.05, **p < 0.01, ***p < 0.001.

To examine potential effects of ERT on the craniofacial phenotype in a more defined and statistically controlled cohort, we utilized the *Arsb* ^*m/m*^ mouse model mimicking human MPS VI. In total we investigated four groups (all at the age of 24 weeks) 1) control *Arsb* ^*+/+*^ mice 2) *Arsb* ^*m/m*^ mice 3) *Arsb* ^*m/m*^ mice receiving ERT starting at 4 weeks of age and 4) *Arsb* ^*m/m*^ mice receiving ERT starting at 12 weeks of age. At first, we analysed the skulls of these mice applying micro-computed tomography (µCT) and decalcified histology. In addition to bone exostoses at the mandibular rim (Figure 2A, white arrow; (Pohl et al., 2018), *Arsb* ^*m/m*^ mice showed exostoses at the zygomatic arch (Figure 2A, red arrows) and the temporal lines (Figure 2A, red arrows). Furthermore, quantifying the mandible and zygomatic arch revealed a significant increase in their volume in *Arsb* ^*m/m*^ mice compared to controls, which could partially be rescued with an early and late start of ERT (Figures 2B,C). In addition, despite open-bite being a prominent skeletal phenotype in MPS VI patients, we did not observe this particular phenotype in *Arsb* ^*m/m*^ mice. However, we detected alteration within the palate of Arsb^+/+^ mice. The palatal shape was changed with an increase in palatal width and height. Only early onset of ERT led to an improvement of this phenotype (Supplementary Fig S1A–D **)**.

**FIGURE 3:**
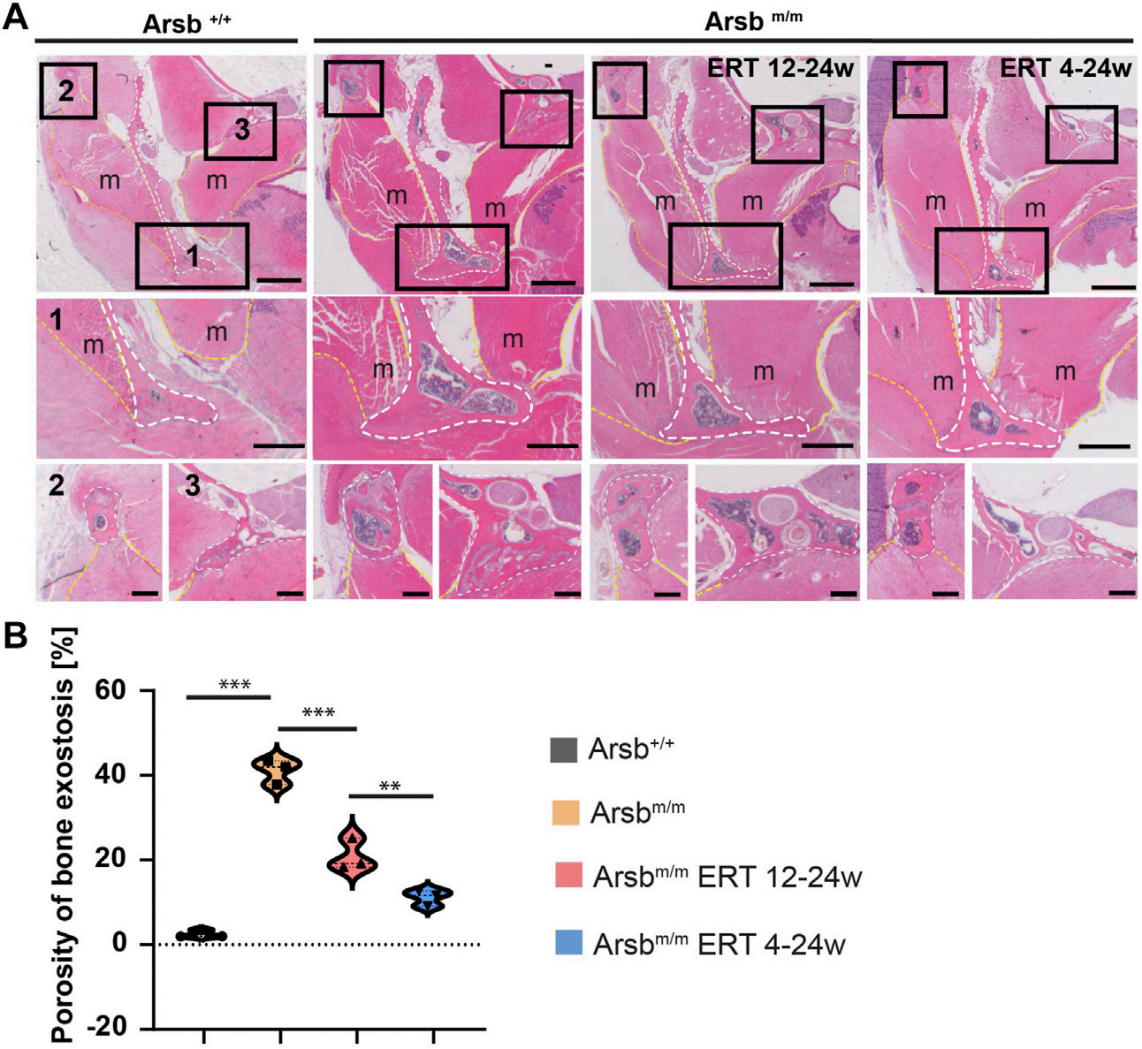
Decalcified sections of the mandibular ridge **(A)** Decalcified sections of the mandibular ridge (white dashed lines) show a higher magnification of the lower mandibular rim 1), zygomatic arch 2), and mastoid process 3). The marked regions show the greater thickness of the bone cortex and increased porosity in ***Arsb*** ^***m/m***^ mice. Bone thickness and porosity are reduced in ***Arsb*** ^***m/m***^ mice with ERT 12-24w and ERT 4-24w. In addition, exostoses are less pronounced in ***Arsb*** ^***m/m***^ ERT 4-24w mice. Scale bars = 1 mm (upper panel), 500 µm (middle panel) and 250 µm (lower panel). m = muscle, TMJ = temporomandibular joints **(B)** Quantification of bone porosity at the inferior border of the mandible. *p < 0.05, **p < 0.01, ***p < 0.001.

To further study the cellular mechanisms of bone exostoses in *Arsb* ^*m/m*^ mice, the skulls were decalcified, sectioned in the coronal plane between the coronoid and condylar processes, and stained with hematoxylin and eosin (Figure 3A). Here, quantitative analyses identified a significant increase in bone porosity in *Arsb* ^*m/m*^ mice compared to control animals (Figure 3B). This increased porosity of the bone was also observed in the zygomatic arc (origin of the masseter muscle) (Figure 3A, Box 2) and the pterygoid process (origin of the medial pterygoid muscle) (Figure 3A, Box 3).

Moreover, in control mice, a protrusion of the bone was visible on the medial side of the mandibular angulus (insertion point of the medial pterygoid muscle) (Figure 3A, white dotted line). In contrast, the mandibles of the *Arsb* ^*m/m*^ mice displayed, in addition to the protrusion on the medial side, protrusion of the bone on the lateral side of the mandibular rim (insertion point of the masseter muscle) (Figure 3A, Box 1).

Together, the present analyses show that 1) the craniofacial phenotype of *Arsb* ^*m/m*^ mice is caused by hyperplasia of the jaw bones, particularly at the origin and insertion of the masseter muscles, and 2) ERT leads to a moderate improvement in mandibular morphology in *Arsb* ^*m/m*^ mice, whereas the thickness and porosity of the arcus zygomaticus and the processus pterygoideus were partially, but significantly corrected.

### Early start of ERT fully recovers alveolar bone loss in *Arsb* ^*m/m*^ mice

The most striking dental symptoms of MPS VI patients include disorders of tooth eruption, such as impacted teeth and follicular tooth eruption cysts (Kantaputra et al., 2014). However, our dental examination of three MPS VI patients revealed significant differences here. While we observed tooth eruption disorders with retention and impaction of permanent teeth in MPS VI patient #1 and #3 (Figures 1G,I), no tooth eruption abnormalities were observed in MPS VI patient #2 (Figure 1H). Interestingly, however, we observed a noticeable alveolar bone loss in this patient (Figure 1H). In fact, the panoramic radiograph showed a horizontal bone loss in the molar region of the maxilla and mandible (Figure 1H, white line).

**FIGURE 4:**
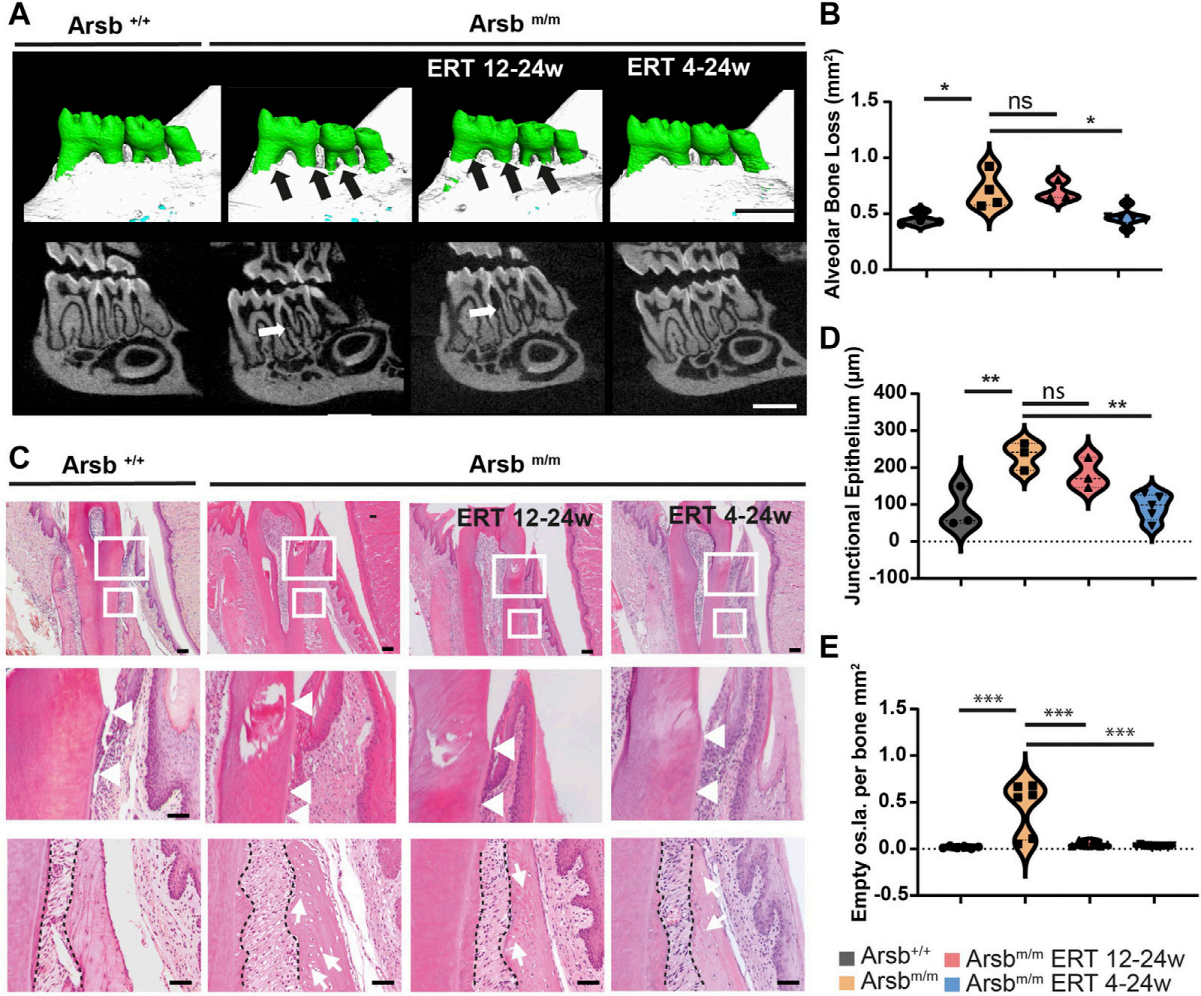
Severe alveolar bone loss and reduced junctional epithelium growth in *Arsb* ^*m/m*^ and late-start ERT *Arsb* ^*m/m*^ mice. **(A)** 3D reconstructions based on μCT scans of the mandibular molars (upper panel). *Arsb* ^*m/m*^ -mice and late-start ERT *Arsb* ^*m/m*^ -mice showing severe alveolar bone loss exposing roots and branches (black arrows). In contrast, control and *Arsb* ^*m/m*^ mice with early start ERT 4-24w show physiological bone levels. Lower panel: μCT cross-sectional view showing low proximal bone level in *Arsb* ^*m/m*^ mice and *Arsb* ^*m/m*^ mice with ERT 12-24 w (white arrows). Scale bar = 1 mm. **(B)** Quantification of alveolar bone loss in control, *Arsb* ^*m/m*^ and early- and late start of ERT *Arsb* ^*m/m*^ mice. **(C)** Decalcified tooth sections stained with hematoxylin and eosin. Higher magnification images of the areas marked by white rectangles representing the junctional epithelium, periodontium, and alveolar bone. The images in the middle panel show apical downward growth of the junctional epithelium in *Arsb* ^*m/m*^ mice, with the cementoenamel junction and the apical end of the junctional epithelium marked with white arrows. In *Arsb* ^*m/m*^ mice with ERT 12-24w and ERT 4-24w, the downward growth was less pronounced. Lower panel: All *Arsb* ^*m/m*^ mice with and without ERT, showed dispersion of the periodontal ligament and hypertrophic osteocytes in the alveolar bone (white arrows). Scale bar = 100 µm. **(D)** Quantification of downward growth of the junctional epithelium. **(E)** Quantification of apparent empty osteocyte gaps per bone of the indicated control and *Arsb* ^*m/m*^ mice. *p < 0.05. **p < 0.01. ***p < 0.001.

To further investigate the pathogenic mechanisms of these dental abnormalities in MPS VI, we next analyzed the teeth and periodontium of *Arsb* ^*m/m*^ mice using µCT (Figure 4A, upper panel). *Arsb* ^*m/m*^ mice showed normal tooth eruption (Figure 4A, lower panel), which did not coincide with the comment impaired tooth eruption in MPS VI patients. This indicated that the murine dentition (monophyodont dentition) might not be suitable for studying this particular aspect. Interestingly, however, a pronounced loss of alveolar bone was detected in *Arsb* ^*m/m*^ mice. The µCT 3D images of *Arsb* ^*m/m*^ mice showed exposed dental roots (Figure 4A, upper panel, black arrows), and the µCT cross-sectional images also demonstrated a reduction of the inter radicular bone, which is usually located at the same level as the floor of the pulp chamber (Figure 4, lower panel, white arrow). To quantify the alveolar bone loss in *Arsb* ^*m/m*^ mice, we measured the visible root surface between the enamel-cement interface and the alveolar bone. Bone loss showed high variability in 24-week-old *Arsb* ^*m/m*^ mice but was significantly increased overall compared to control mice of the same age (Figure 4B). *Arsb* ^*m/m*^ mice with late start of ERT (*Arsb* ^*m/m*^ ERT 12-24w, showing a similar degree of alveolar bone loss (Figures 4A,B). In contrast, early start of ERT in *Arsb* ^*m/m*^ mice (*Arsb* ^*m/m*^ ERT 4-24w) completely corrected the alveolar bone loss (Figure 4A, bottom row, and Figure 4B).

To analyze the loss of alveolar bone in *Arsb* ^*m/m*^ mice in more detail, we next prepared decalcified sections followed by staining with hematoxylin and eosin of the teeth. During microscopic analysis, we also examined the junctional epithelium, a specialized epithelial layer that connects the connective tissue of the gingiva to the tooth surface. In control mice, the junctional epithelium was localized at the enamel-cement junction and extended on average 87 µm apically (Figures 4C,D, white triangles). In contrast, in *Arsb* ^*m/m*^ mice, we observed a 170% longer apically migrated junctional epithelium (Figures 4C,D). In addition to bone loss, this elongation and apical migration is another sign of periodontium loss in *Arsb* ^*m/m*^ mice. While the junctional epithelium was still elongated in *Arsb* ^*m/m*^ mice with late start of ERT, complete normalization was achieved with early start of ERT (Figures 4C,D).

Furthermore, we observed that the osteocyte lacunae in the alveolar bone appeared to be enlarged (Figure 4C, third panel), which have been reported for the tibia and vertebral body of *Arsb* ^*m/m*^ mice (Hendrickx et al., 2020). Quantification of the number of these empty osteocyte lacunae in the alveolar bone revealed, despite a large variance, an overall significant increase in *Arsb* ^*m/m*^ mice compared to control mice (Figure 4E). After both early and late start ERT, almost a complete normalization of the number of empty osteocyte lacunae was achieved (Figure 4E).

Taken together these results show, that the earlier ERT is started in *Arsb* ^*m/m*^ mice the better the degradation of the periodontium is prevented.

### Arsb^m/m^ mice display a change in condyle morphology

Hypoplasia of the maxillary condyle is one of the most prominent craniofacial symptoms in MPS VI. (Roberts et al., 1984; de Santana Sarmento et al., 2015; Schmid-Herrmann et al., 2021). Although this symptomatology is usually not associated with pain, the temporomandibular joint changes can lead to a restricted mouth opening and a relatively vertical facial growth (Figure 1J) (Fonseca et al., 2014). Indeed, we observed marked hypoplasia of the temporomandibular joint condyles (Figures 1G,I) and a vertical growth pattern in our MPS VI patients on a cephalometric image (Figure 1J).

**FIGURE 5:**
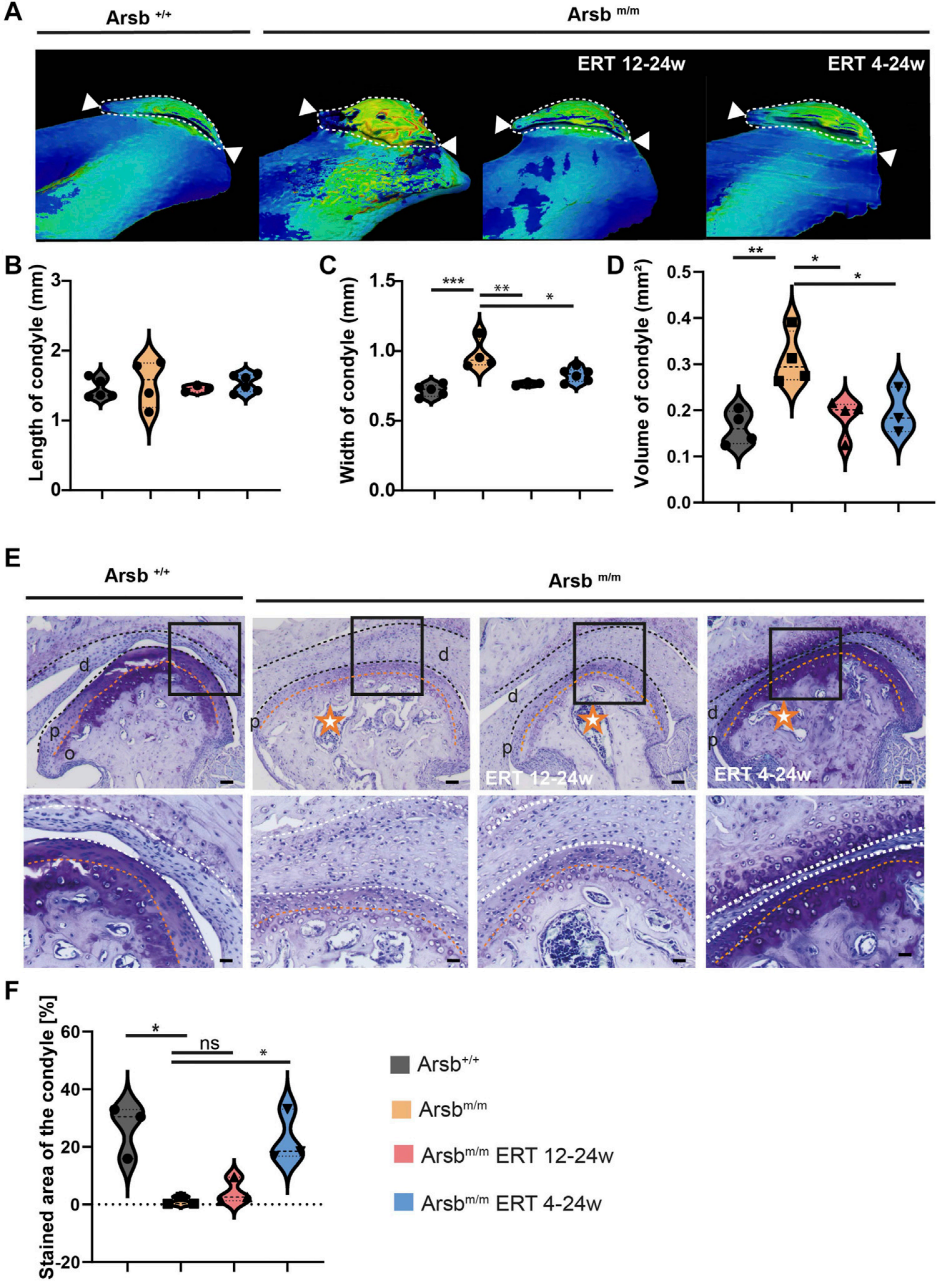
Dysmorphic condyle in *Arsb* ^*m/m*^ mice and *Arsb* ^*m/m*^ mice with ERT from 12 weeks of age. **(A)** 3D segmentation and wall thickness analysis of micro-CT scans of the condyle of 24-weeks control, *Arsb* ^*m/m*^ -mice, *Arsb* ^*m/m*^ -mice with ERT from 12–24 weeks, and ERT from 4–24 weeks. *Arsb* ^*m/m*^ -mice and *Arsb* ^*m/m*^ -mice with ERT from 12 weeks show bone exostoses and deformative pathologies of the mandibular condyle. Scale bar = 100 µm. **(B)** Quantification of condylar length. **(C)** Quantification of condylar width. **(D)** Quantification of condyle volume **(E)** Toluidine blue staining showing intense pigmentation of cartilage revealing physiological proteoglycan levels in control and *Arsb* ^*m/m*^ mice with ERT from 4 weeks. In contrast, *Arsb* ^*m/m*^ mice without therapy and ERT show faded staining from 12 weeks. Middle panel: At higher magnification, *Arsb* ^*m/m*^ mice show bone porosity (orange stars) and a pathological condyle shape. In addition, the cartilage-bone line in *Arsb* ^*m/m*^ mice is irregularly shaped and marked with an orange dashed line. Lower panel: The superficial chondrocytes in the condyles and the chondrocytes in the articular disc of control mice show physiological size and shape (green arrows). Chondrocytes in *Arsb* ^*m/m*^ mice appear markedly hypertrophic, including vacuolization (orange arrows). In contrast, *Arsb* ^*m/m*^ mice treated with ERT from week four onwards have fewer hypertrophic chondrocytes (green arrows), almost equal to those of control mice. Scale bars = 100, 50, and 25 µm (from top to bottom). d = discus articularis, p = proliferating zone, o = sclerosing zone **(F)** Quantification of toluidine blue-stained area within the condyle *p < 0.05. **p < 0.01. ***p < 0.001.

We next segmented the condyles from the μCT scans to investigate whether *Arsb* ^*m/m*^ mice show similar changes. In control mice, we observed a physiologically round and convex shape of the mandibular condyle (Figure 5A). In contrast, *Arsb* ^*m/m*^ mice showed resorptions on the condylar surface and bone exostoses in the distal region of the condyle. While condyle morphology still appeared altered in *Arsb* ^*m/m*^ mice with late start ERT, early start ERT led to a typical bone structure and a physiologically round-convex condyle shape (Figure 5A). To further support these findings, we measured the length, width, and volume of the mandibular condyle using µCT imaging. The length of the condyle in *Arsb* ^*m/m*^ mice revealed no significant changes compared to control animals (Figure 5B). In contrast a 34% increase in the condylar width was observed in *Arsb* ^*m/m*^ mice (Figure 5C). Moreover, we found significant increases of the condyle volume in *Arsb* ^*m/m*^ mice (Figure 5D). Interestingly, both early and late start ERT resulted in almost complete normalization of condylar width and volume in *Arsb* ^*m/m*^ mice (Figures 5C,D). Although the temporomandibular joint changes in *Arsb* ^*m/m*^ mice only partially reflect the clinical phenotype of MPS VI patients, our results demonstrate that ERT can lead to an improvement of condyle morphology.

### Altered composition of glycosaminoglycans in the temporomandibular joint cartilage of *Arsb* ^*m/m*^ -mice

Glycosaminoglycans provide the articular cartilage with its resistance to pressure loads by binding water molecules (Buckwalter and Mankin, 1998). To analyze the relative amounts and distribution of GAGs of the temporomandibular joints, we sectioned 24-week-old control, *Arsb* ^*m/m*^ mice with and without ERT treatment in the anterodorsal direction followed by staining the temporomandibular joint with toluidine blue to visualize GAGs. While in articular cartilage of control mice a physiological intense staining was observed, *Arsb* ^*m/m*^ mice and *Arsb* ^*m/m*^ mice with late start ERT showed significantly reduced staining of articular cartilage. In contrast, *Arsb* ^*m/m*^ mice with early start ERT displayed the physiological staining as in control mice (Figures 5E,F). Next, we used safranin O staining to visualize GAGs, which stains the temporomandibular joint of control mice red while the bone tissue appeared blue-green. In contrast to control mice, *Arsb* ^*m/m*^ mice without ERT showed altered staining of GAG in the cartilage. Here, only early start ERT could restore the physiological staining of articular cartilage (Supplementary Figure S2A). In summary, the GAG content in temporomandibular joints of *Arsb* ^*m/m*^ mice was reduced in the cartilage tissue, which could be reversed by early start ERT only.

## Discussion

In this work, the craniofacial system and dentoalveolar structures of Arsb-deficient mice, a mouse model for the human MPS VI disease, were investigated by contact radiography, micro-computed tomography, and histology. Our results show that the deficiency of arylsulfatase B in *Arsb* ^*m/m*^ mice leads to alterations of the jawbone, alveolar bone, and temporomandibular joints that largely mirror the craniofacial and dental phenotype of MPS VI patients. Furthermore, we demonstrate that early administration of rhARSB replacement therapy can prevent the development of most craniofacial pathologies.

### Jaw exostoses are associated with the origin and insertion points of the masticatory musculature in *Arsb* ^*m/m*^ mice

In previous studies, we showed that *Arsb* ^*m/m*^ mice exhibit exostoses at the mandibular rim, which are less pronounced by an early start of ERT (Pohl et al., 2018; Hendrickx et al., 2020). Similarly, we observed exostoses at the mandibular rim in a mouse model for mucolipidosis type II (MLII) (Koehne et al., 2016) and MPS I (Kuehn et al., 2015). In this study, we could show for the first time by μCT and histological analyses that these exostoses are associated with the attachment point of the masticatory muscles. In particular, our histological analysis revealed hyperplasia of the jaw bone at the origin of the masseter muscle (the arcus zygomaticus), and the origin of the medial pterygoid muscle (the pterygoid process). A systemically increased bone mass in *Arsb* ^*m/m*^ mice, including the mandible, may explain these hyperplasia in the jaw bone (Pohl et al., 2018; Hendrickx et al., 2020). However, it appears to be more likely that in *Arsb* ^*m/m*^ mice, deposits of non-degraded GAG in the masseter muscles lead to increased traction on the jaw bones and subsequently to reactive hyperplasia of the jaw bone at the origin as well as attachments of the jaw muscles. This pathomechanism (i. e. secondary bone growth due to increased muscle traction) would also explain why similar exostoses have been observed in the MLII mouse model (Koehne et al., 2016), despite the prevailing osteopenic bone phenotype. However, further studies are needed to analyze changes in soft tissues of MPS patients, e.g. by MRI (Koehne et al., 2018).

In this context, it should be emphasized that changes in the face are often among the first symptoms noticed in MPS patients and are stigmatizing for the patient group. Therefore, a precise analysis of the facial changes (e.g., on 3D photographs (face scans)) could support predictions on the success of the enzyme replacement therapy. On the other hand, our experimental data confirm that complete normalization of facial morphology cannot be expected even with an early start of ERT. The effects of early administration of ERT in MPS VI on craniofacial changes, however, have not been included in respective clinical trials (Horovitz et al., 2021).

### Early initiation of ERT can prevent alveolar bone loss in Arsb-deficient mice

Although disorders of tooth development are regularly reported in MPS VI (Smith et al., 1995; Hirst et al., 2021), the teeth of *Arsb* ^*m/m*^ mice have not been studied so far. We could show that tooth development and eruption occur without disruption in *Arsb* ^*m/m*^ mice, although in MPS VI patients, impaired tooth eruption accompanied by the development of tooth cysts, and tooth displacement, have been observed. A reason for this could be the difference in the murine dentition. Unlike humans, mice have a monophyodont dentition, and permanent molars erupt at an early age of 2–3 weeks. Here the accumulation of GAGs did not advance enough to affect tooth eruption. This phenomenon is in line with humans as the dental phenotype is only established in permanent teeth, as children exhibit mostly normal eruption of their primary teeth. Only later eruption of the permanent tooth is affected (James et al., 2012). This monophyodont dentition might indicate that the murine dentition is therefore unsuitable for studying this particular aspect. Interestingly however, our studies shows that the periodontium in *Arsb* ^*m/m*^ mice showed a significant bone loss. Moreover, we observed a significantly elongated junctional epithelium, another sign of periodontal disease. This is of particular interest as it has been shown that glycosaminoglycans such as dermatan sulfate and chondroitin sulfate are present in the periodontal ligament and provide adhesion of the epithelium to the tooth (Kurylo et al., 2016). It can therefore be assumed that these glycosaminoglycans are responsible for maintaining the connection between the tooth root and the alveolar bone. (Fujii and Hirabayashi, 1999). It is possible that the synthesis/secretion of specific proteoglycans containing dermatan sulphate and chondroitin sulphate is reduced in the junctional epithelium of *Arsb* ^*m/m*^ mice. This would also explain the observed bone resorption around the tooth, as the loss of periodontal fibre connection inevitably leads to a loss of alveolar bone. Furthermore, it could be possible that the enlarged cells and consequently thickened periodontal ligament displace the bone. In addition, patients with other MPS subtypes (such as MPS I and II also show periodontal changes (Antunes, Nogueira et al., 2013). Here it would be interesting to investigate subtype-specific periodontal diseases in MPS patients and how they can be differentiated from classical bacterial periodontitis, also concerning a possible improvement by enzyme replacement.

### The temporomandibular joint is altered in *Arsb* ^*m/m*^ mice

Changes in the temporomandibular joint are characteristic of MPS VI patients and manifest clinically mainly through limited mobility of the mandible (Cavaleiro et al., 2013; Palmucci et al., 2013; de Almeida-Barros et al., 2018). Here, it should be emphasized, that hypoplasia of the mandibular condyle in *Arsb* ^*m/m*^ mice would have been expected based on human clinical findings. However, *Arsb* ^*m/m*^ mice with and without enzyme replacement therapy tended to have an irregular condylar shape and surface. The condyle width was significantly greater in *Arsb* ^*m/m*^ mice compared to control mice. Thus, although *Arsb* ^*m/m*^ mice did not phenotypically fully reflect the joint pathology of the patients, it should be noted that *Arsb* ^*m/m*^ mice with early initiation of enzyme replacement therapy at 4 weeks of age exhibited standard condylar shape. Thus, an earlier start of enzyme replacement therapy might prevent these degenerative changes.

On a histological level, the articular cartilage of *Arsb* ^*m/m*^ mice also showed changes compared to control mice. This is consistent with the finding describing that Arsb deficiency in cats leads to severe disorganization in the epiphyseal groove, which includes greatly enlarged cartilage cells with membranous inclusions resulting from an accumulation of glycosaminoglycans (Haskins et al., 1980). In addition, Hendrickx et al. described in *Arsb* ^*m/m*^ mice that chondrocytes in the articular cartilage of the acetabulum were enlarged and that the uptake of rhARSB in cultured chondrocytes of *Arsb* ^*m/m*^ mice was significantly reduced (Hendrickx et al., 2020). This would also explain why no complete normalization of cell morphology in articular cartilage was observed in our histological analyses of *Arsb* ^*m/m*^ mice with enzyme replacement therapy. Here the cause could be two-fold 1) the extracellular matrix within the bone with its particularly high SO_4_ content is hindering the M_6_P-containing enzymes to reach the cell surface and 2) the relatively poor blood supply to cartilage tissue. It is, therefore, worth mentioning that in an animal experiment, a significant reduction in the enlargement of chondrocytes in various joints (knee, shoulder, elbow) was observed by intra-articular injection of rhARSB (Auclair et al., 2006). A similar effect would be conceivable with the intra-articular injection of rhARSB in the temporomandibular joint.

Most striking histologically, however, was that in toluidine blue and safranin O staining, the proteoglycans in the temporomandibular joint of *Arsb* ^*m/m*^ mice and *Arsb* ^*m/m*^ mice with late start ERT stained weaker compared to control mice. This finding is interesting because GAGs typically accumulate in almost all other tissues and organs due to the absence of ARSB. However, 1) we cannot differentiate here between extra- and intracellular GAGs and 2) it is also possible that non-degraded GAGs cannot be stained correctly by the two staining methods. In any case, an early start of ERT showed complete normalization of cartilage staining.

Overall, the results show that enzyme replacement therapy over 12–24 weeks has a limited effect on cartilage and thus on joints in the mouse model. Therefore, further research is needed to understand the underlying mechanisms and develop targeted local therapeutic interventions (e.g. an intra-articular injection) that could be used alongside systemic enzyme replacement therapy. For example, a study in rats could show that a combination of ERT and an anti-inflammatory drug (anti-TNF-α) led to a significant improvement in cartilage and bone (Eliyahu et al., 2011). In this work, however, an early start of enzyme replacement therapy also showed a significant protective effect in the TMJ. Therefore, an early start of ERT is recommended for the temporomandibular joint as well.

Furthermore, due to the specific location of the bone exostoses in the skull (inside of the mandible, proc. coronoideus, arcus zygomaticus, and the upper edge of the temporal fossa), it is of interest that these structures represent the origin and attachment of the masticatory muscles in the mouse. Although we did not detect any significant change in muscle anatomy, a possible change in shape/growth could explain the coarsened facial features and bilateral thickening of the mandible in some MPS VI patients. However, this requires further in-depth validation and experimentation.

In summary, this work shows that *Arsb* ^*m/m*^ mice largely resemble MPS VI patients in craniofacial and dental findings and that the mouse model, therefore, allows conclusions to be drawn about the clinical treatment of patients. The results in the mouse model show that an early start of enzyme replacement therapy can achieve the best therapeutic effect, even with an almost complete recovery in some tissues. As such, early enzyme replacement therapy also benefits the craniofacial system.

## Material and methods

### Laboratory animals

The experimental animals originated from previous experimental projects (Pohl et al., 2018; Hendrickx et al., 2020) and were available in fixed form at the Institute of Osteology and Biomechanics. The animal experiments were approved by the Authority for Health and Consumer Protection of the Free and Hanseatic City of Hamburg (43/15 and G14/068, Org529). Control mice (Arsb^+/+^) and MPS VI mice (*Arsb* ^*m/m*^) were studied at 24 weeks of age. In addition, 24-week-old *Arsb* ^*m/m*^ mice that received enzyme replacement therapy (ERT) with rhARSB (Naglazyme, BioMarin, Novato, California, United States) intravenously from 4 to 12 weeks of age were analyzed. For this purpose, a single weekly dose of 1 mg/kg body weight with a volume of 150 µl was administered.

### Micro-computed tomography

The skulls of the experimental animals were scanned with a micro-computed tomography (µCT-40, SCANCO Medical, Brüttisellen, Switzerland). The three-dimensional analysis was performed with the integrated device software. Avizo 3D-Pro was used for semi-automatic segmentation of the skull and mandible and for volume measurements.

### Histology

All samples to be sectioned and histologically examined were placed in an embedding cassette. Subsequently, the samples were placed in a decalcifying solution (USEDECALC, MEDITE Medical GmbH, Burgdorf, Germany) for 14–17 days. The solution was changed every 3 days. After decalcification, the samples were dehydrated overnight in an auto technical unit. Within 12 h, the samples were dehydrated in an ascending alcohol series, and fixative and tissue fluid were replaced with paraffin. Sections of 4 µm thickness were cut with a microtome (Supercut 2050, Reichert-Jung, Leica Microsystems GmbH, Wetzlar, Germany) and stained with hematoxylin-eosin, toluidine blue, or safranin O. The sections were then cut with a microscope. For this purpose, the sections were deparaffinized in a xylene bath (3 × 5 min). The samples were rehydrated in descending alcohol series for 2 min each, followed by a short wash in distilled water. They were then stained according to standard protocols. The stained sections were then rinsed with distilled water and dehydrated in an ascending alcohol series. Before covering with Eukitt (ORSAtec GmbH, Bobingen, Germany), infiltration with xylene was performed in three successive baths for 5 min each.

### Histological evaluation

A microscope (Axio Scope. A1, Carl Zeiss Microscopy GmbH, Jena, Germany) and a photo camera (Axiocam, Carl Zeiss Microscopy GmbH, Jena, Germany) were used for image acquisition. For the histological analysis, each measurement was performed blind, i.e., only the examiner knew the assignment of the individual samples to avoid distortions.

### Cartilage thickness and condylar porosity

Both parameters were measured and analyzed with OsteoMeasure (OsteoMetrics Inc., Decatur, Georgia, United States). Decalcified stained sections were viewed under a microscope. Cartilage was marked manually, and mean thickness was calculated by the software. For calculating the porosity of the mandibular condyle bone and areas without bone were selected manually. Then the bone-absent area was divided by bone area to calculate a percentage value.

### Alveolar bone loss and junctional epithelium

Measurements were performed with ImageJ (National Institutes of Health, Bethesda, Maryland, United States). In micro-CT images, the root area between the cementoenamel junction and alveolar bone was manually marked and calculated by the software. For calculating the length of absent periodontal junctional epithelium, images of stained sections were first calibrated, and then the distance between C.E.J. and the beginning of the attached junctional epithelium was calculated.

### Palatal length and thickness

Two distances were determined to measure the different extent in palatal coronal width. First, palatal width was determined as the interval between the mesio-palatal cusps of the first molars. Furthermore, the total expansion of the os palatinum was measured by the distance between the most lateral points of the processus pyramidalis. The thickness was further analysed by defining the broadest expansion of the crista nasalis at the level of the mesio-palatal cusps of the first molars.

### Statistical evaluation

Graphs were plotted, and statistical analysis of the data was performed using GraphPad PRISM 8 software (GraphPad Software Inc., San Diego, California, United States). A one-factor analysis of variance (ANOVA) with Tukey’s posthoc test was used for statistical analysis. The significance value or *p*-value was marked with asterisks as follows: *p* ≤ 0.05 = *, *p* ≤ 0.01 = **, and *p* ≤ 0.001 = ***.

## Data Availability

The original contributions presented in the study are included in the article/Supplementary Material, further inquiries can be directed to the corresponding authors.
