# Supplementary figures and images for: Early enzyme replacement therapy prevents dental and craniofacial abnormalities in a mouse model of mucopolysaccharidosis type VI
Source: Front Physiol. 2022 Sep 21;13:998039. doi: 10.3389/fphys.2022.998039 (PMC9532570; doi:10.3389/fphys.2022.998039)

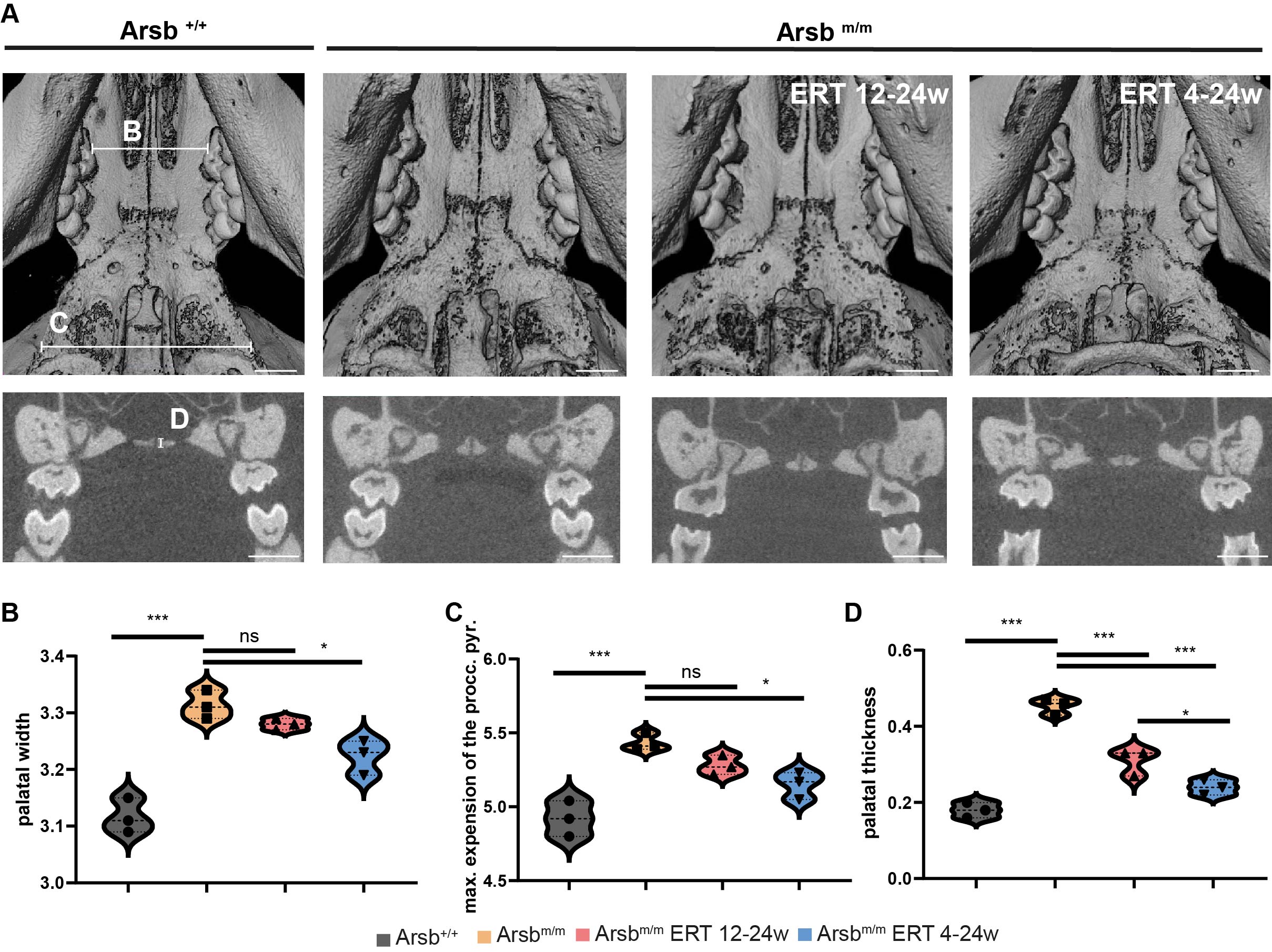

Supplement: Supplementary file 1 [file Image1.jpeg]

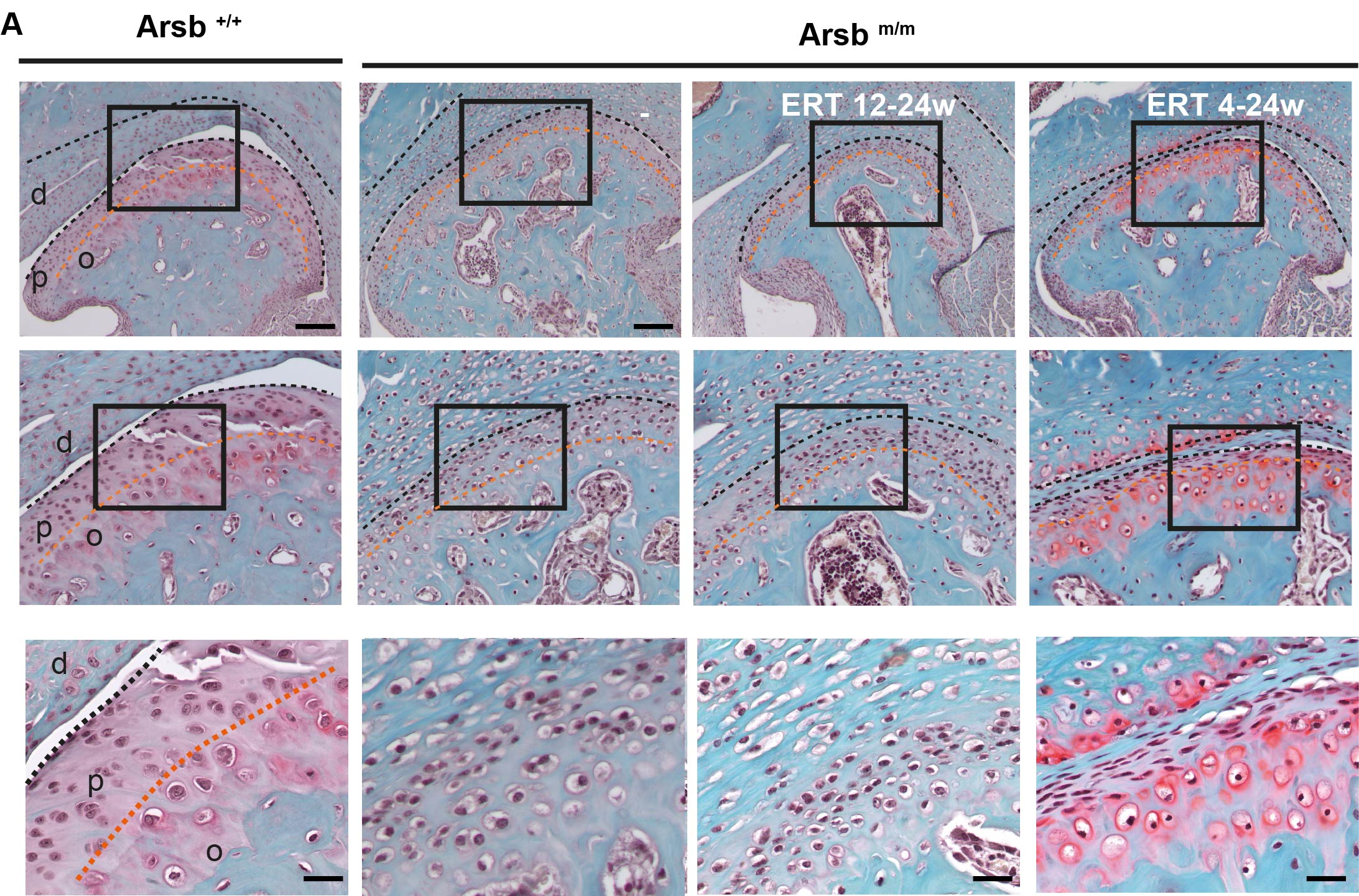

Supplement: Supplementary file 2 [file Image2.jpeg]
